# Aquatic therapy for boys with Duchenne muscular dystrophy (DMD): an external pilot randomised controlled trial

**DOI:** 10.1186/s40814-017-0132-0

**Published:** 2017-03-27

**Authors:** Daniel Hind, James Parkin, Victoria Whitworth, Saleema Rex, Tracey Young, Lisa Hampson, Jennie Sheehan, Chin Maguire, Hannah Cantrill, Elaine Scott, Heather Epps, Marion Main, Michelle Geary, Heather McMurchie, Lindsey Pallant, Daniel Woods, Jennifer Freeman, Ellen Lee, Michelle Eagle, Tracey Willis, Francesco Muntoni, Peter Baxter

**Affiliations:** 1 Sheffield Clinical Trials Research Unit, University of Sheffield, Sheffield, UK; 2 School of Health and Related Research, Sheffield, UK; 3 Department of Mathematics and Statistics, University of Lancaster, Lancaster, UK; 4 Evelina London Childrens Hospital, Guy’s and St Thomas’ NHS Foundation Trust, London, UK; 5 Aquaepps, Dorking, Surrey UK; 6 Dubowitz Neuromuscular Centre (DNC), The UCL Great Ormond Street Institute of Child Health and Great Ormond Street Hospital for Children NHS Foundation Trust, London, UK; 7 Children’s Therapy Department, University Hospital Southampton NHS Foundation Trust, Southampton, UK; 8 Paediatric Physiotherapy, Heart of England NHS Foundation Trust, Birmingham, UK; 9 Regional Paediatric Neuromuscular Team, Leeds Teaching Hospitals NHS Trust, Leeds, UK; 10 Paediatric Neurology, Sheffield Children’s Hospital, Sheffield, UK; 11 Leeds Institute of Health Sciences, University of Leeds, Leeds, UK; 12 Newcastle Muscle Centre, Newcastle University, Newcastle, UK; 13 The Oswestry Inherited Neuromuscular Service, The Robert Jones and Agnes Hunt, Orthopaedic Hospital NHS Foundation Trust, Oswestry, UK

**Keywords:** Duchenne muscular dystrophy, Aquatic therapy, Hydrotherapy, Physical therapy, Pilot study, Feasibility study

## Abstract

**Background:**

Standard treatment of Duchenne muscular dystrophy (DMD) includes regular physiotherapy. There are no data to show whether adding aquatic therapy (AT) to land-based exercises helps maintain motor function. We assessed the feasibility of recruiting and collecting data from boys with DMD in a parallel-group pilot randomised trial (primary objective), also assessing how intervention and trial procedures work.

**Methods:**

Ambulant boys with DMD aged 7–16 years established on steroids, with North Star Ambulatory Assessment (NSAA) score ≥8, who were able to complete a 10-m walk test without aids or assistance, were randomly allocated (1:1) to 6 months of either optimised land-based exercises 4 to 6 days/week, defined by local community physiotherapists, or the same 4 days/week plus AT 2 days/week. Those unable to commit to a programme, with >20% variation between NSAA scores 4 weeks apart, or contraindications to AT were excluded.

The main outcome measures included feasibility of recruiting 40 participants in 6 months from six UK centres, clinical outcomes including NSAA, independent assessment of treatment optimisation, participant/therapist views on acceptability of intervention and research protocols, value of information (VoI) analysis and cost-impact analysis.

**Results:**

Over 6 months, 348 boys were screened: most lived too far from centres or were enrolled in other trials; 12 (30% of the targets) were randomised to AT (*n* = 8) or control (*n* = 4). The mean change in NSAA at 6 months was −5.5 (SD 7.8) in the control arm and −2.8 (SD 4.1) in the AT arm. Harms included fatigue in two boys, pain in one. Physiotherapists and parents valued AT but believed it should be delivered in community settings. Randomisation was unattractive to families, who had already decided that AT was useful and who often preferred to enrol in drug studies. The AT prescription was considered to be optimised for three boys, with other boys given programmes that were too extensive and insufficiently focused. Recruitment was insufficient for VoI analysis.

**Conclusions:**

Neither a UK-based RCT of AT nor a twice weekly AT therapy delivered at tertiary centres is feasible. Our study will help in the optimisation of AT service provision and the design of future research.

**Trial registration:**

ISRCTN41002956

**Electronic supplementary material:**

The online version of this article (doi:10.1186/s40814-017-0132-0) contains supplementary material, which is available to authorized users.

## Background

International guidelines for the multidisciplinary management of people with Duchenne muscular dystrophy (DMD) recommend regular physical therapy, including stretching and positioning regimens 4–6 days/week to maintain joint ranges, and submaximum aerobic exercise/activity to avoid disuse atrophy. Exercises in water are highly recommended. They note that the evidence base for these recommendations is weak and do not detail specific therapy interventions or dosage, nor do they discuss aquatic therapy [1].

Aquatic therapy (AT), sometimes also called hydrotherapy, is defined by the UK Aquatic Association of Chartered Physiotherapists (ATACP) as “a therapy programme utilising the properties of water, designed by a suitability qualified physiotherapist specifically for an individual to improve function, carried out by appropriately trained personnel, ideally in a purpose built, and suitably heated hydrotherapy pool” [2]. Subsequently, hydrotherapy was specified to be a programme designed by a physiotherapist but implemented by carers or teaching assistants, while aquatic physiotherapy is delivered by a specialist physiotherapist [3]. There are limited data on the effectiveness of AT in general [4–6], and none in people with DMD. Our study addressed a 2012 commission from the UK National Institute for Health Research (NIHR) Health Technology Assessment (HTA) programme for a feasibility study (HTA 12/144/04). The specific objective was to collect data that would tell us whether it was feasible to run a full-scale trial, assessing the clinical effectiveness of AT in maintaining physical function in people with Duchenne muscular dystrophy. The principal focus of this paper is the feasibility of a full-scale research study. With the debate about terminology unresolved [7, 8], we present this data as an external pilot trial [9], supplemented by a distillation of qualitative research findings, reported more fully elsewhere, in line with guidance [10]. Although some reference to patient and professional views on the feasibility of the intervention are integral to understand the feasibility of the trial, this topic is addressed more fully elsewhere.

## Methods

### Trial design

The protocol for this pilot trial, which was developed with patient and public involvement, is available on the NIHR website [11]. The design was an external pilot trial for a parallel-group, open-labelled, individually randomised controlled trial with a 1:1 allocation ratio incorporating nested qualitative research. This report is compliant with the Consolidated Standards of Reporting Trials (CONSORT: see Additional file 1) [12].

### Participants

Between October 2014 and June 2015, we planned to recruit 40 boys from six UK centres with a paediatric neuro-muscular service (Birmingham, Great Ormond Street Hospital London, Leeds, Oswestry, Southampton, Sheffield). Medical notes were reviewed to select ambulant boys aged 7–16 years with genetically confirmed DMD, North Star Ambulatory Assessment (NSAA) score of ≥ 8, established on corticosteroid therapy, that is, a patient has been treated with prednisolone or deflazacort for at least 6 months with no major change in drug, dosage or frequency for at least 3 months before the initial assessment. The major changes were defined as (1) frequency of change from daily to alternate day or other non-daily regimen (or vice versa) and (2) dose increase in line with weight is acceptable. The other change is an exclusion criterion: (3) drug changes from prednisolone to deflazacort (or vice versa). Potential participants were assessed to determine if they were able to complete a 10-m walk test with no walking aids or assistance as part of the North Star Ambulatory Assessment (Item 2). We excluded those who were involved in another clinical trial; had more than 20% variation between screening and baseline NSAA scores; were unable to commit to the programme of twice weekly AT for 6 months; and/or had any absolute contraindications or precautions to AT listed in the study protocol [11].

Potentially eligible boys and their carers were invited to an initial consent appointment. After assessment, written assent from the boys and consent from their carers, they were randomly allocated in a 1:1 ratio by a centralised web-based randomisation system provided by the Sheffield Clinical Trials Research Unit (CTRU) to AT plus land-based therapy (LBT) or LBT alone. Allocation concealment was achieved by ensuring the participant identifier was entered by the physiotherapist, following which the allocation was revealed; no member of the study team had access to the randomisation schedule during recruitment.

### Interventions

Standardised AT was prescribed and delivered by an AT-trained physiotherapist (with specialist knowledge of DMD), based on the study protocols detailed in a manual, in 30-min sessions twice weekly in an NHS pool with a temperature of 34–36 °C (Additional file 2). Standardised LBT stretches and exercises were prescribed by a specialist physiotherapist at the baseline appointment, based on the study protocols detailed in a manual (Additional file 3). With the aim of reducing missing data, the research physiotherapists sent reminders to participants who had not sent back the LBT proforma. A maximum of three reminder letters were sent to each participant over the course of the trial. Those randomised to receive AT were asked to perform LBT on four of the other 5 days of the week, while those in the control group were asked to perform LBT 6 days/week.

### Outcomes

The primary outcome was the feasibility of recruitment of 40 participants within 6 months from six centres. Additional feasibility outcomes were a decision on the primary endpoint for a subsequent larger trial; the number and characteristics of eligible participants who were approached for the study; the number of participants randomised, withdrawn, and lost to follow-up; the number of participants who discontinued AT and were included and excluded from analysis with reasons; the recruitment rate; reasons for refused consent; participant attrition rates and reasons; data completeness; feasibility of recruiting participating sites and estimation of the costs; participant views on acceptability of research procedures and intervention; physiotherapist views on the intervention/research protocol and perceived contamination of the control group; and intervention optimisation.

The following clinical data were collected for all participants: 6-min walk distance (6MWD) [13]; North Star Ambulatory Assessment (NSAA) [14, 15]; forced vital capacity (FVC); Child Health Utility 9D Index (CHU9D)—health state utility [16]; carer quality of life (CarerQoL—carer burden) questionnaire [17]; activity limitations measure (ACTIVLIM) [18]; and a health and social care resource-use questionnaire. Parents were also requested to return, in stamped addressed envelopes, diaries recording the LBT stretches/exercises performed over the previous 4 weeks. In participants allocated to AT, the Wong-Baker visual analogue scale for pain and the Children’s OMNI Scale of Perceived Exertion [19] were assessed before and after each AT session. The therapists also recorded attendance as well as the AT stretches and exercises performed.

### Blinding

The participants, physiotherapists and physicians were not blinded to treatment allocation. The statisticians and health economist were blinded to treatment allocation until the statistical analysis plan was agreed and signed.

### Sample size

The sample size for this external pilot trial was based on a recommended minimum of 30 participants (15 per group) for feasibility objectives involving parameter estimation [20]. Assuming a drop-out rate at 6 months of 20%, we set a target of randomising at least 40 participants (20 per group). While this decision was principally informed by the need to calculate a sample size for a full-scale study [9], we believed the recruitment of 40 boys in 6 months might indicate the feasibility of a trial of *n* = 100 to 150 in UK centres alone, given a longer recruitment window that would still be acceptable to funding bodies (1 to 2 years). Initial sample size estimates for a full-scale study, working in a frequentist framework, centred around a total *n* of 610 (80% power, two-sided, to detect 5%—18 m—difference in the 6MWD with 10% drop-out at 12 months). We were confident that, by using Bayesian methods [21] and selecting an meaningful outcome measure with the right measurement properties, we could reduce this sample size considerably.

### Feasibility criterion

This pilot aimed to recruit 40 children in 6 months and deliver AT to 20 of them. If this objective success criterion was met, then we could deem a full-scale study potentially feasible. Other feasibility outcomes did not involve objective stop-go (success) criteria but provided a basis for improving the research procedures.

### Statistical methods

Quantitative analysts (SR and TY) remained blind to treatment allocation until after the statistical analysis plan was finalised, the database locked and the data review completed. The intention to treat population (ITT) included all patients who were consented and randomised. This was the primary analysis set, and unless stated otherwise, all endpoints are summarised for the ITT population. Depending on the distribution of the data, continuous variables (e.g., age) were summarised by either the mean and standard deviation or the median and interquartile range (IQR). AT adherence was assessed by the number and percentage of AT sessions attended, with mean (SD), median (IQR) and minimum–maximum numbers. LBT adherence was measured by the number of days on which the prescribed exercises were performed and the percentage of the prescribed exercises that were performed on across the total number of days on which exercise adherence was recorded.

Descriptive statistics (mean differences between groups and 95% confidence intervals (CIs)) were derived for clinical outcomes. Categorical outcomes are presented as the difference between groups in the percentages in each category, together with 95% CIs. Available clinical outcomes at 6 months are presented for the ITT set, by group and overall. For continuous outcomes, we present change from baseline by group and overall.

We estimated the cost of the AT intervention to families and the NHS using information from the quantitative and qualitative components of the study. Data completeness was a fidelity outcome of the study. A sensitivity analysis involving imputation for missing data was not performed. Prior to database lock, all missing data was queried with staff at centres and data management. Questionnaires were scored only when all the items that made up a domain were complete for each patient and carer questionnaires.

To further assess the feasibility of a full-scale RCT evaluating the clinical effectiveness of AT, we proposed group sequential designs for such a trial that would use conventional ‘frequentist’ statistics to perform definitive tests of hypotheses. Testing at the 2.5% one-sided significance level, adaptive designs were calibrated to have 80% power to detect a target difference of 9 points in a primary endpoint of linearised NSAA score at 6 months, assuming scores are normally distributed with standard deviation 15 points, values which look realistic given the results in [22]. Expected and maximum sample sizes of designs were recorded. Also considered were Bayesian single-stage designs measuring a primary endpoint of change from baseline at 6 months in linearised NSAA score with prior distributions informed by pilot study data. Properties of Bayesian designs based on pragmatic sample sizes were computed via simulation, assuming that on completion the Bayesian trial would declare AT plus land-based exercises superior to land-based exercises alone if the posterior probability of a benefit of AT exceeded 0.9.

### Intervention optimisation

An independent rater (IR) reviewed patient records (medical, social and school history), as well as data from baseline assessments, parent-completed LBT exercise diaries, therapist-completed AT session data and attendance logs, to determine the levels of treatment optimisation.

### Qualitative research

Trial management group (TMG) meeting minutes and email communications helped assess barriers to implementation of the trial and the intervention. Semi-structured interviews with participants and parents (*n* = 8 boys) and health professionals (*n* = 8) were completed to gather views on acceptability of the AT intervention and research protocols. All interviews were audio recorded and transcribed. Transcripts were coded in NVivo with analysis completed using a framework analysis. The methods and results of this qualitative research component will be reported fully in the NIHR journals library (12/144/04).

### Patient and public involvement

Co-authors JP, a young man with DMD, and VW, his mother and former carer, were involved throughout, in the design of both the AT intervention and the study, in the qualitative research analysis and in drafting outputs.

### Ethical approval

This trial received ethical approval from Research Ethics Committee East of England—Cambridge South (14/EE/0204).

## Results

### Study implementation

Of the initial six partner centres, two could not participate (Liverpool, Newcastle), due to a lack of pool availability or problems accessing excess treatment costs. In the UK, treatment costs are those that fall upon healthcare commissioners rather than grant-awarding bodies, where the cost of an experimental treatment exceeds the cost of the standard care [23]. The other four centres consented a total of ten boys. In view of this, further 11 UK centres were approached of which two participated (Southampton and Oswestry), consenting an additional three boys. The other eight centres did not participate for the following reasons: inability to obtain regulatory approval in time, treatment costs, lack of eligible participants within a reasonable travelling distance, pool availability and/or lack of staff.

### Recruitment and baselines

**Fig. 1:**
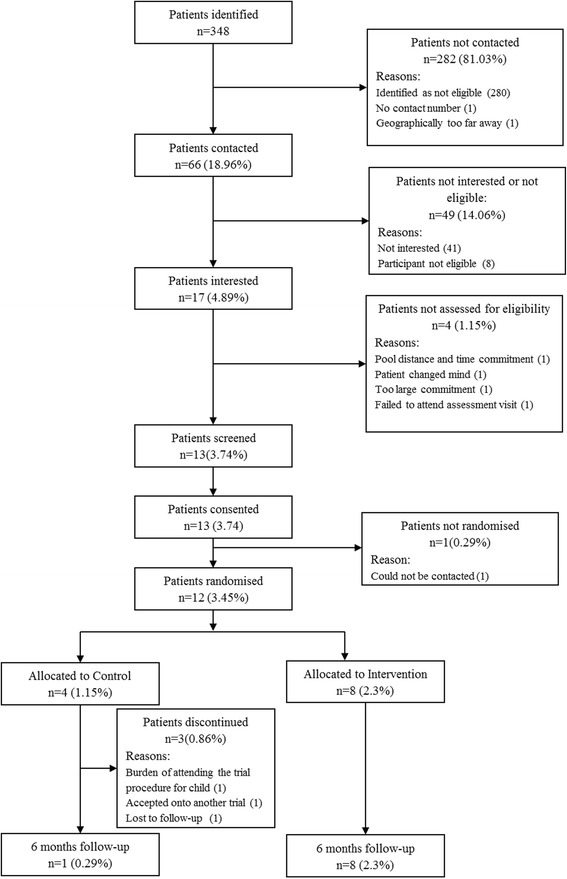
Participant flow diagram

Recruitment occurred between October 2014 and June 2015, and the participants were to be followed up 26 weeks (±2 weeks) from the date of randomisation. Overall, 348 boys with DMD were reviewed for potential eligibility across the six participating sites. Of these, 17 were interested and eligible after an initial telephone contact, 13 screened and consented, and 12 randomised to the study: 8 to the AT group and four to the control group (Fig. 1, Table 1). One withdrew before randomisation to participate in another clinical trial. The trial ended when the planned accrual period elapsed.

**Table 1:** Demographics

	Control	Intervention	Total
Age			
*n*	4	8	12
Mean (SD)	9.8 (2.5)	8.0 (0.9)	8.6 (1.7)
Median (IQR)	9.5 (8.0, 11.5)	8.0 (7.5, 8.0)	8.0 (7.5, 9.5)
Min, max	7, 13	7, 10	7, 13
Ethnicity			
English/Welsh/Scottish/Northern Irish/British	3 (75.0%)	2 (25.0%)	5 (41.7%)
Any other White backgrounds	1 (25.0%)	2 (25.0%)	3 (25.0%)
Indian	0	1 (12.5%)	1 (8.3%)
Any other Asian backgrounds	0	2 (25.0%)	2 (16.7%)
Any other mixed/multiple ethnic backgrounds	0	1 (12.5%)	1 (8.3%)
Others (specify)			
Korean	0	1	1
Filipino	0	1	1
Polish	1	2	3
Weight (kg)			
*n*	2	5	7
Mean (SD)	25.550 (2.616)	26.480 (4.572)	26.214 (3.910)
Median (IQR)	25.550 (23.700, 27.400)	26.500 (23.800, 26.600)	26.500 (23.700, 27.400)
Min, max	23.70, 27.40	21.70, 33.80	21.70, 33.80
Height (cm)			
*n*	2	5	7
Mean (SD)	117.000 (0.849)	119.960 (6.280)	119.114 (5.339)
Median (IQR)	117.000 (116.400,117.600)	121.000 (114.600,121.200)	117.600 (114.600,121.200)
Min, max	116.40,117.60	113.70,129.30	113.70,129.30

### Numbers analysed, outcomes and estimation

**Fig. 2:**
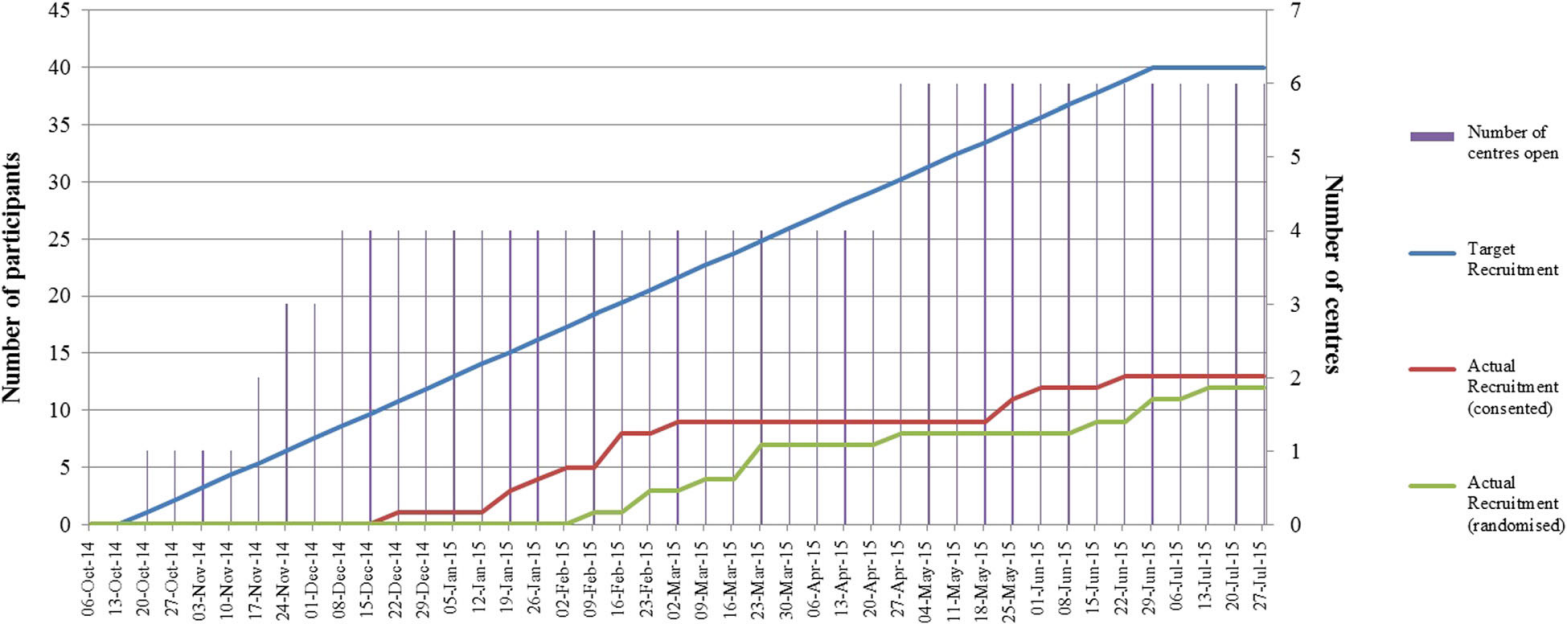
Number of participants randomised by months

**Fig. 3:**
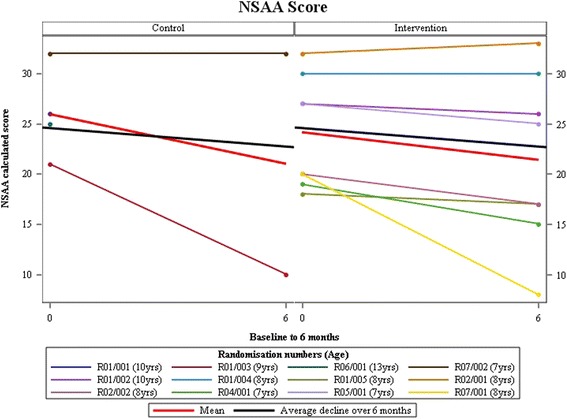
North Star Ambulatory Assessment (NSAA) scores

**Fig. 4:**
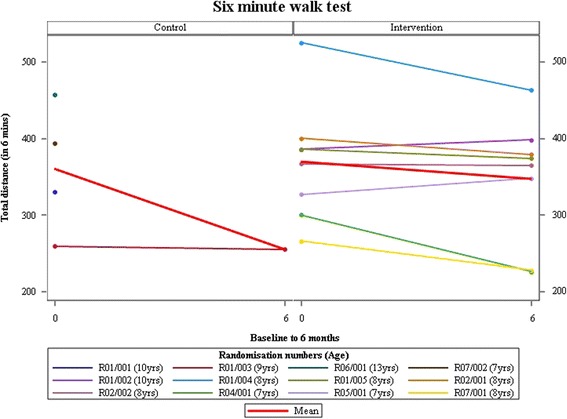
Change in 6mWD over 6 months

At the 26 week follow-up visit, eight AT and one control participants contributed 6MWT data (Fig. 2). In the control group, two participants withdrew before completing because of the burden of attending the trial procedure for child and acceptance onto another trial, respectively, while another was lost to follow-up (Table 2). NSAA data was available for one of these. Primary and secondary clinical outcomes showed a small difference favouring AT, but the numbers are too small to allow significance testing (Table 3, Figs. 3 and 4). No serious adverse events and 15 adverse events were reported: 10 falls, two influenza immunisations, and one each for chest infections, sleep hypo-ventilation and delayed onset muscle soreness. Five participants recruited to the AT group showed no pain or fatigue before or after AT on the Wong-Baker and Children’s OMNI scales while the other three reported increases in one or both (Table 4).

**Table 2:** External pilot trial completion summary

Site	Date initiated	Consented	Randomised	6-month visit (completed)	Withdrew consent	Lost to follow-up	Other withdrawn
R01	24/10/2014	5	5	4	1	0	0
R02	27/11/2014	2	2	2	0	0	0
R04	11/12/2014	1	1	1	0	0	0
R05	19/11/2014	2	1	1	0	0	1
R06	28/04/2015	1	1	0	1	0	0
R07	29/04/2015	2	2	1	0	1	0

**Table 3:** Summary of outcomes for primary and secondary outcomes by intervention group

		Control				Intervention			
Outcome measure	Follow-up	*n*	Mean(SD)	Median(IQR)	Min–max	*n*	Mean(SD)	Median(IQR)	Min–max
6 min total distance	Baseline	4	360(84.98)	362(294.5–425.5)	259–457	8	369.63(78.39)	376.5(313.5–393)	266–525
	6 months	1	255	255(255–255)	255–255	8	347.63(81.88)	369.5(288–388.5)	226–463
NSAA score	Consent	4	25.75(3.4)	26.5(23.5–28)	21–29	8	23.38(5.93)	22.5(18.5–29)	16–31
	Baseline	4	26(4.55)	25.5(23–29)	21–32	8	24.13(5.49)	23.5(19.5–28.5)	18–32
	6 months	2	21(15.56)	21(10–32)	10–32	8	21.38(8.47)	21(16–28)	8–33
FVC absolute	Consent	4	1.42(0.21)	1.43(1.27–1.57)	1.16–1.66	7	1.52(0.2)	1.5(1.27–1.74)	1.27–1.78
	Baseline	2	1.29(0.21)	1.29(1.14–1.43)	1.14–1.43	5	1.34(0.19)	1.39(1.3–1.48)	1.03–1.5
	6 months	0				5	1.33(0.42)	1.52(1–1.6)	0.77–1.76
FVC percent	Consent	2	90.5(9.19)	90.5(84–97)	84–97	4	97.25(11.62)	97.5(89–105.5)	83–111
	Baseline	2	88.5(7.78)	88.5(83–94)	83–94	5	90.8(17.09)	91(79–101)	70–113
	6 months	0				5	83.8(22.57)	88(68–93)	56–114
CHU utility value	Baseline	3	0.92(0.07)	0.89(0.87–1)	0.87–1	8	0.77(0.23)	0.88(0.59–0.94)	0.39–0.96
	6 months	1	0.95	0.95(0.95–0.95)	0.95–0.95	8	0.87(0.09)	0.87(0.82–0.95)	0.71–1
CarerQol score	Baseline	3	31.27(10.37)	26.2(24.4–43.2)	24.4–43.2	7	40.6(22.9)	29.3(24.4–59.4)	19.7–81.6
	6 months	1	50.1	50.1(50.1–50.1)	50.1–50.1	7	51.27(6.78)	50.1(48.8–50.4)	44–65.8
ACTIVLIM patient score	Baseline	3	32.67(9.71)	35(22–41)	22–41	8	30.38(7.58)	32(26.5–34)	17–41
	6 months	1	21	21(21–21)	21–21	8	26.88(6.36)	26(22.5–31.5)	19–36
ACTIVLIM patient measure	Baseline	3	2.98(2.62)	4.15(−0.02–4.82)	−0.02–4.82	8	2.1(1.37)	2.22(1.61–2.78)	−0.6–4.15
	6 months	1	0.18	0.18(0.18–0.18)	0.18–0.18	8	1.29(1.13)	1.48(0.4–2.1)	−0.35–2.73

NSAA is scored from 0 to 34 where higher scores represent higher function. CarerQol is scored from 0 to 100 where higher scores represent better care situation. CHU values range from 0.33(worst state) to 1 (perfect health). Higher ACTIVLIM scores represent higher activity

**Table 4:** Summary of clinical outcomes for the intervention group only

				Before				After				Increase			
ID	Scoring	Attendance status	Session counts	*n*	Mean(SD)	Median(IQR)	Min–max	*n*	Mean(SD)	Median(IQR)	Min–max	*n*	Mean(SD)	Median(IQR)	Min–max
R01/002	Wong–Baker	Full	28	21	0.43(1.03)	0(0–0)	0–4	21	0.38(0.8)	0(0–0)	0–2	21	−0.05(0.5)	0(0–0)	−2–1
	Wong–Baker	Partial	1	1	0	0(0–0)	0–0	1	0	0(0–0)	0–0	1	0	0(0–0)	0–0
	OMNI	Full	28	10	2.5(0.53)	2.5(2–3)	2–3	27	2.44(0.8)	2(2–3)	1–4	10	0.5(0.71)	0(0–1)	0–2
	OMNI	Partial	1	0				1	3	3(3–3)	3–3	0			
	N/A	Did not attend	16												
R01/004	Wong–Baker	Full	29	21	0.1(0.44)	0(0–0)	0–2	21	0.1(0.44)	0(0–0)	0–2	21	0(0)	0(0–0)	0–0
	OMNI	Full	29	10	2.7(0.48)	3(2–3)	2–3	28	2.43(0.79)	3(2–3)	1–4	10	0.3(0.48)	0(0–1)	0–1
	N/A	Did not attend	16												
R01/005	Wong–Baker	Full	29	28	0.04(0.19)	0(0–0)	0–1	28	0.04(0.19)	0(0–0)	0–1	28	0(0)	0(0–0)	0–0
	Wong–Baker	Partial	1	0				0				0			
	OMNI	Full	29	10	0.9(1.6)	0(0–1)	0–5	28	0.75(1.14)	0(0–1)	0–5	10	0.1(0.32)	0(0–0)	0–1
	OMNI	Partial	1	0				0				0			
	N/A	Did not attend	15												
R02/001	Wong–Baker	Full	17	16	0.06(0.25)	0(0–0)	0–1	16	0.19(0.54)	0(0–0)	0–2	16	0.13(0.5)	0(0–0)	0–2
	Wong–Baker	Partial	6	6	0.33(0.82)	0(0–0)	0–2	6	0(0)	0(0–0)	0–0	6	−0.33(0.82)	0(0–0)	−2–0
	OMNI	Full	17	15	0.2(0.41)	0(0–0)	0–1	16	3.13(0.96)	3(3–4)	1–5	15	3.07(0.7)	3(3–4)	2–4
	OMNI	Partial	6	6	0.5(1.22)	0(0–0)	0–3	6	1.83(1.6)	2(0–3)	0–4	6	1.33(2.5)	2(0–3)	−3–4
	N/A	Did not attend	29												
R02/002	Wong–Baker	Full	16	16	0.25(0.68)	0(0–0)	0–2	16	1.06(1.44)	0(0–2)	0–4	16	0.81(1.22)	0(0–2)	0–4
	Wong–Baker	Partial	2	2	0(0)	0(0–0)	0–0	2	1(1.41)	1(0–2)	0–2	2	1(1.41)	1(0–2)	0–2
	OMNI	Full	16	14	0.5(0.85)	0(0–1)	0–2	16	1.88(1.75)	2(0–2.5)	0–6	14	1.07(1.82)	1.5(0–2)	−2–4
	OMNI	Partial	2	2	0(0)	0(0–0)	0–0	2	4(0)	4(4–4)	4–4	2	4(0)	4(4–4)	4–4
	N/A	Did not attend	34												
R04/001	Wong–Baker	Full	28	28	0(0)	0(0–0)	0–0	26	0(0)	0(0–0)	0–0	26	0(0)	0(0–0)	0–0
	OMNI	Full	28	0				28	0(0)	0(0–0)	0–0	0			
	N/A	Did not attend	10												
R05/001	Wong–Baker	Full	15	15	0(0)	0(0–0)	0–0	15	0(0)	0(0–0)	0–0	15	0(0)	0(0–0)	0–0
	Wong–Baker	Partial	1	1	0	0(0–0)	0–0	1	0	0(0–0)	0–0	1	0	0(0–0)	0–0
	OMNI	Full	15	2	0.5(0.71)	0.5(0–1)	0–1	15	0.33(0.82)	0(0–0)	0–3	2	1(1.41)	1(0–2)	0–2
	OMNI	Partial	1	0				1	0	0(0–0)	0–0	0			
	N/A	Did not attend	12												
R07/001	Wong–Baker	Full	26	26	1.65(1.65)	2(0–2)	0–5	26	2.65(2)	2(2–4)	0–7	26	1(1.67)	0.5(0–2)	−2–4
	Wong–Baker	Partial	4	4	3.5(4.12)	3(0–7)	0–8	4	3.75(4.35)	3.5(0–7.5)	0–8	4	0.25(0.5)	0(0–0.5)	0–1
	OMNI	Full	26	8	4(0.76)	4(3.5–4.5)	3–5	26	3.85(2.54)	4(2–5)	0–8	8	2.38(1.6)	2(1–3.5)	1–5
	OMNI	Partial	4	2	6(1.41)	6(5–7)	5–7	4	7.5(1.91)	7(6–9)	6–10	2	2(1.41)	2(1–3)	1–3
	N/A	Did not attend	14												

Wong–Baker is scored from 0 to 10 with higher scores representing worse pain. OMNI is scored from 0 to 10 with higher scores representing more tiredness

### Intervention implementation

The median (range) time between randomisation and initiation of AT was 47 (7–211) days and mean 63 days. Of the 349 sessions scheduled where data were available, 203 (58.2%) of the expected sessions took place and 146 (41.8%) did not. Cancellation of AT sessions was due to healthcare provider factors in 47% and participant/family factors in 43%, with 10% unaccounted for.

### Intervention optimsation

AT and LBT adherence summaries are given in Tables 5 and 6, and a summary of AT attendance by participant in Table **7**. In general, there was a low return of LBT forms (*n* = 4/12) but in those returned, a good adherence to the prescription.

**Table 5:** Intervention adherence: aquatic therapy session summary

	NSAA score			Total no. of sessions	Total no. of stretches competed/total no. of stretches prescribed per session (%)			
Randomisation	Consent	Baseline	6 months	*N*	Median	Min	Max	Mean
R01/002	25	27	26	29	33.33	14.81	48.15	30.99
R01/004	31	30	30	29	29.17	12.5	52.17	28.91
R01/005	16	18	17	29	40.91	8.7	68.18	38.63
R02/001	31	32	33	23	92.86	42.86	100	86.77
R02/002	18	20	17	18	93.33	46.67	100	90
R04/001	20	19	15	28	41.21	21.21	100	51.55
R05/001	27	27	25	16	51.67	26.67	63.64	49.76
R07/001	19	20	8	30	100	36.36	100	91.52

**Table 6:** Intervention adherence: land-based therapy week summary

Randomisation		NSAA score			Total no. of sessions	Total no. of stretches with data/total no. of stretches prescribed per session (%)			
ID	Group	Consent	Baseline	6 months	*N*	Median	Min	Max	Mean
R01/005	Research intervention	16	18	17	27	66.67	58.33	100	74.69
R02/001	Research intervention	31	32	33	24	81.87	30.77	100	72.16
R02/002	Research intervention	18	20	17	24	100	87.5	100	97.92
R07/001	Research intervention	19	20	8	20	100	80	100	93.67

**Table 7:** AT attendance summary by participant

Randomisation number	Actual sessions attended	Actual sessions not attended	Patient factors	Pool factors	Unknown	Available sessions*	Percent attendance based on available pool
R01/002	29	16	5	10	1	34	85
R01/004	29	16	4	10	2	33	88
R01/005	30	15	5	10	0	35	86
R02/001	23	29	14	14	1	37	62
R02/002	18	34	20	14	0	38	47
R04/001	28	10	5	5	0	33	85
R05/001	16	12	6	6	0	22	73
R07/001	30	14	8	6	0	38	79

*Due to late-starting, some participants could not have completed 52 sessions by the time of study closure.

The independent reviewer (IR) judged the AT prescription as good for three participants, variable by session for two and poor for three. For 4/8, they were realistic and achievable. The number of exercises prescribed per session varied between 4 and 27. Prescription compliance varied between 20 and 40% for three participants and 70 and 90 for five. 5/8 participants completed additional non-prescribed exercises. The IR considered exercises not to be appropriately prioritised in three cases. In part, this resulted from a difference in understanding regarding whether the AT manual was to be used as a menu of possible exercises or as a whole. LBT prescriptions were considered appropriate and achievable for the four AT participants who returned the data. However, after intervention by the community physiotherapist midway through the study, the number of exercises increased and compliance decreased in three. The prescribed number of exercises ranged from 5 to 13 per day.

### Feasibility of the intervention

In five interviews, parents or boys reported some improvement was experienced after AT; in one case, the parent’s view was discordant with that of the boy and physiotherapist. Less tangible physical benefits, such as loosening of muscles, were valued by parents and most physiotherapists, many of whom also noted improvements in water confidence, social confidence or well-being. Two boys reported fatigue, one transient pain. Two parents volunteered that they had sustained back injures through delivering LBT. Parents were highly satisfied with their aquatic therapists, highlighting their skill in making exercise fun for boys. It was generally agreed that the twice weekly AT programme was too burdensome, with an average journey of 15 miles and competing pressures on time. Notwithstanding this, there was enthusiasm for continuing once weekly hydrotherapy at more convenient locations, and gaps in attendance (see above) did not present a pattern that suggested decreased commitment over time.

At two trusts, the physiotherapists challenged the value of the AT, highlighting that it required diversion of scarce resources from other activities. While the training emphasised that AT prescriptions should focus on the individual’s capability and needs, many therapists thought they had to prescribe stretches suitable for dry land regardless of relevance. Some were dissatisfied with the intervention as a result. NHS reorganisation, following the Health and Social Care Act 2012, made it difficult to identify/communicate with community AT/physiotherapy services [22] in order to gain the appropriate NHS R&D permissions, leading us to run the study and deliver the intervention through specialist centres. However, all physiotherapists believed that AT should be delivered in community rather than specialist settings but valued the new skills they had acquired and felt that they would advocate regular and intensive AT where resources allowed. Participating trusts discussed subcontracting out AT delivery to therapists at centres, nearer to participant homes (minutes, TMG, 7 July 2014, 19 January 2015). But, with a rare and geographically dispersed population, we would have had to seek approvals from and contracted with almost as many community trusts as we recruited participants and would have had to train as many interventionists.

Over 6 months, the estimated direct NHS costs ranged from £1970 to £2734 and the societal costs ranged from £2541 to £3775, based on attendance. Financial pressures and opportunity costs were noted by the physiotherapists as making the service difficult to justify.

### Feasibility of the trial

The physiotherapists and parents believed recruitment to the trial was difficult because industry trials were more attractive to parents and co-enrolment is prohibited by UK research ethics committees. They believed that participant retention was contingent on randomisation to AT, as confirmed by the drop-out rate observed in our control arm. Recruitment was particularly difficult where access to AT was already good.

That was one of the worries that by partaking in this would it stop him being eligible for another trial because, as great as this is, as a parent, you’re always online seeing what NICE are going to approve. (Parent)

I think any patient wanting to, to participate in this trial would basically have wanted to be in the, err in the hydro arm or not bother. (Physiotherapist)

The difficulty was there’s so many trials going on at the moment for Duchenne that it was difficult to get appropriate patients. And because a lot of ours get hydrotherapy anyway, yeah, it was difficult. (Physiotherapist)

Another major barrier to recruitment was the distance from the pool. Partly due to therapist availability and partly due to a national reorganisation of health service provision and funding at the time, we could not easily access community pools, with the result being that many possible participants lived at least 20 miles away with round-journey times of 2 hours or more. This proved too great a burden for many families to face twice weekly for 6 months, especially given that, under the Department of Health rules for funding research, travel costs for interventions count as treatment costs and cannot be reimbursed from a research grant [23].

There were several parents who… couldn’t make that commitment to twice a week to driving into central [area name]. There were other parents who said that if it was more local, if they didn’t have siblings, if they didn’t feel the child would be tired after school, after coming all the way into central [area name]… Generally parents were quite keen on the idea… they would’ve [participated] had the situation been easier for them. (Physiotherapist)

Where boys remembered randomisation, they were negative about it, and only two parents were positive. Some physiotherapists believed the eligibility criteria should have been relaxed to allow enrolment of younger or more disabled boys. The physiotherapists and parents found the completion of intervention documentation for fidelity purposes burdensome, and the physiotherapists would require more administrative support for any future research. They believed that a future trial would need long-term follow-up and mechanistic outcomes in order to identify a treatment effect. Three families reported the questionnaire battery as too burdensome, and two complained about the vagueness of questions on the instruments used to evaluate health-related quality of life. The 6MWD is typically the most common primary outcome in trials for ambulant boys with DMD. As in other studies, concerns about the feasibility of this assessment were raised during the study, due to difficulties finding 30-m long corridors and staff trained to complete the assessment (Minutes, Trial Management Group, 15 Sep 2014, 03 Nov 2014).

Table 8 provides a data completeness summary; this shows the number of participants the data were available for per group and the percentage of data available, with 100% being all data we expected. The NSAA score was the only outcome available for all participants expected at each time point, taking into account participant withdrawals. The questionnaire completion rates are provided in Table 9 with the ACTIVLIM being the only questionnaire with missing data in the control group and the ACTIVLIM and CareQOL having missing data in the intervention group.

**Table 8:** Data completeness

Scoring	Follow-up time point	Control (*N* = 4) *n* (col %)	Intervention (*N* = 8) *n* (col %)	Overall (*N* = 12) *n* (col %)
NSAA score	Consent	4 (100)	8 (100)	12 (100)
	Baseline	4 (100)	8 (100)	12 (100)
	6 months	2 (100)	8 (100)	10 (100)
FVC absolute	Consent	4 (100)	7 (88)	11 (92)
	Baseline	2 (50)	5 (63)	7 (58)
	6 months	0 (0)	5 (63)	5 (56)
FVC % predicted for height	Consent	2 (50)	4 (50)	6 (50)
	Baseline	2 (50)	5 (63)	7 (58)
	6 months	0 (0)	5 (63)	5 (56)
6-min total distance	Consent	0 (0)	0 (0)	0 (0)
	Baseline	4 (100)	8 (100)	12 (100)
	6 months	1 (100)	8 (100)	9 (100)
CHU utility value	Consent	0 (0)	0 (0)	0 (0)
	Baseline	3 (75)	8 (100)	11 (92)
	6 months	1 (100)	8 (100)	9 (100)
CarerQOL score	Consent	0 (0)	0 (0)	0 (0)
	Baseline	3 (75)	7 (88)	10 (83)
	6 months	1 (100)	7 (88)	8 (89)
CarerQOL happy VAS	Consent	0 (0)	0 (0)	0 (0)
	Baseline	3 (75)	8 (100)	11 (92)
	6 months	1 (100)	8 (100)	9 (100)
ACTIVLIM patient score	Consent	0 (0)	0 (0)	0 (0)
	Baseline	3 (75)	8 (100)	11 (92)
	6 months	1 (100)	8 (100)	9 (100)
ACTIVLIM patient measure	Consent	0 (0)	0 (0)	0 (0)
	Baseline	3 (75)	8 (100)	11 (92)
	6 months	1 (100)	8 (100)	9 (100)

**Table 9:** Questionnaire completion (%)

		Control		Intervention		Overall	
Scoring	Follow-up time point	Min–max	Median	Min–max	Median	Min–max	Median
NSAA score	Consent	100–100	100	100–100	100	100–100	100
	Baseline	100–100	100	100–100	100	100–100	100
	6 months	100–100	100	100–100	100	100–100	100
CHU	Baseline	100–100	100	100–100	100	100–100	100
	6 months	100–100	100	100–100	100	100–100	100
ACTIVLIM	Baseline	81.82–100	100	63.64–100	95.45	63.64–100	100
	6 months	81.82–81.82	81.82	72.73–100	81.82	72.73–100	81.82
CarerQOL	Baseline	100–100	100	85.71–100	100	85.71–100	100
	6 months	100–100	100	71.43–100	100	71.43–100	100

A full-scale RCT following a group sequential design incorporating up to two interim analyses permitting early stopping for success or futility according to the ρ-family error spending criteria (with parameter *ρ* = 2; see Chapter 7 of [24]) would recruit the following: on average, 74.4 patients if AT plus land-based exercises are truly superior to land-based exercises alone; on average, 62.1 patients if both interventions are equivalent; and a maximum of 93.3 patients. These sample sizes are likely to be prohibitive in light of the data from this pilot trial. Rather than make definitive comparisons between interventions, a Bayesian trial based on a pragmatic sample size would settle for the less ambitious objective of improving our understanding of the effectiveness of AT. Assuming a vague uniform (0, 100) prior for the response SD and an independent normal prior for the treatment difference with mean 5.375 and variance 213.2, Table 10 lists the probability that a full-scale Bayesian RCT would conclude declaring AT plus land-based exercises superior to land-based exercises alone, for various sample sizes and data scenarios. The prior for the treatment difference is based on pilot study data: the mean is the difference between the sample mean changes from baseline in linearised NSAA score on each trial arm; the prior SD is twice the standard error of the difference in sample means to down-weight the contribution of the pilot information.

**Table 10:** Results are based on 10,000 simulations

True treatment difference	Probability future RCT declares AT plus optimised land-based exercises superior to optimised land-based exercises alone			
	*N* = 20	*N* = 30	*N* = 40	*N* = 60
0	0.10	0.10	0.11	0.10
*δ*/2	0.27	0.33	0.38	0.39
*δ*	0.52	0.64	0.73	0.75
3*δ*/2	0.75	0.88	0.94	0.93

*N* is the total sample size divided equally between interventions. Primary endpoint of a future Bayesian trial would be change from baseline at 6 months in linearised NSAA score, and the ‘true treatment effect’ refers to the underlying difference between average outcomes on each intervention. Future trial data are simulated according to the model $\widehat{\theta}$ ~ *N*(θ, 4σ^2^/*N*) and s^2^ ~ (σ^2^/*N*) *χ*_*N*_^2^_− 2_ setting *σ* = 15 and *δ* = 9, where $\widehat{\theta}$ is the difference between sample mean outcomes on each treatment arm (and *θ* is the true treatment difference) and *s* ^2^ is the pooled sample response variance

## Discussion

This pilot trial has demonstrated that a standard full-scale randomised controlled trial would not be feasible, as originally planned, in the UK alone. Our data should help in planning future studies of both AT and LBT in DMD. The majority of UK centres we approached could not participate for a variety of reasons. Variability in pool availability and AT provision has been highlighted as a problem by a national charity [25]. NHS treatment costs (see the “Results” section) have long been known as a barrier to the timely completion of publicly funded research in the UK, and our study also suffered as a result of the lack of nationally implementable guidelines [26, 27].

In the participating centres, which included some of the largest in the UK, the majority of boys assessed did not meet our eligibility criteria (Fig. 1). In those who were ambulant and therefore eligible, many did not participate, mostly because of current or anticipated participation in other trials. In the time between planning our study and implementing it, several trials started, the existence of which we had not been aware. Although the protocols of some, such as the DMD Heart Protection Study (ISRCTN50395346), permitted co-enrollment, we were unable to obtain ethics committee approval for co-enrollment to our own study.

All those randomised to the AT group completed the study, but three of the four in the control group did not, for the same reasons that prevented initial recruitment, as discussed above. Randomising as individuals meant that one family with more than one eligible boy had two boys in the AT group and another in the control group.

Our study was not powered to detect clinically important differences when AT was added to LBT. The intended sample of 40 would have permitted power calculations for a future full-scale trial [20, 28]. There have not been any previous reported studies of AT in DMD. Due to problems associated with the 6MWD, the routinely collected NSAA [15, 29, 30] appears to be the most feasible outcome for any future full-scale trials. This could minimise data collection costs and loss of information due to the NSAA being routinely collected for DMD patients at clinic appointments. Rare disease groups can benefit from the use of the cohort multiple randomised controlled trial design (cmRCT) or Trials Within Cohorts (TWiCs) [31], which uses staged-informed consent to overcome drop-out and other problems associated with interventions in the absence of equipoise [32]. Pragmatic trials of physiotherapy interventions in DMD could be more feasible if the disease registries such as the NorthStar Project were able to adopt cmRCT/TWiCs functions in the future.

When planning this study, we found that there were no agreed validated standardised protocols for either AT or LBT, either in terms of what to do or the dosage (frequency and duration). We therefore created our own manuals for both (reported separately). Conventionally, in the UK, AT is given as 6-week blocks but we chose a higher dosage to ensure that any benefit would not be missed through under-dosage. Independent review of the intervention optimisation showed that the dose varied and that the protocols were not always appropriately applied. In general, AT was well tolerated but in some, it caused pain or fatigue.

For LBT, there was some evidence of an inverse relationship between dosage and compliance. In addition, the poor return of LBT data suggests poor compliance. One mother of a family with four affected boys told us she never performed home LBT as she did not have the time or resources.

We have insufficient evidence to demonstrate whether AT provides value for money in terms of quality-adjusted life years gained. The direct (health system) costs (£1970 to £2734) are less than haemodialysis for children aged over 6 months (£10,296 to £46,352 for three sessions per week) or specialist paediatric services for cystic fibrosis (£2554 to £24,809 over 6 months) [33]. Based on experience accrued before and since the trial was designed, service users and therapists have proposed a number of ways that services could be made cheaper and more feasible.

1. Health systems could increase the number of pool sessions offered by the health system by combining access to children with other neuromuscular conditions in the pool and requiring parents to be in the pool while a physiotherapist rotates between clients.
2. Health systems should enable parents to safely deliver targeted AT techniques, through in-pool training and diagram sheets. Give them responsibility to access warm water pools between courses provided by the health system.
3. Health systems and voluntary sector organisations could enable parents to link with each other, in order to share the documented high costs of warm-water pool hire and conduct AT exercises regularly at their convenience [25]. In some places, this ‘hydrotherapy club’ model has been enhanced by limited support from qualified and volunteer student physiotherapists, without being cost-prohibitive [34].
4. For boys who are in special schools, access is already often better where technical instructors, physical therapy assistants and other staff members can deliver AT based on our manuals with limited oversight by health system physiotherapists.

Both the qualitative research findings and the patient and public involvement underline how much families value AT in participation terms and dedicated parents are willing to negotiate complex systems in search of better access. We end this discussion section with a statement from our service user representatives, James and Victoria:

The ideal is open-ended, weekly therapist-led AT, but the current service is sporadic four- to six-week blocks. James found he had to rebuild all his confidence when coming back to therapist-led AT after breaks in service. Sustaining water confidence and self-esteem is very important to self-management. If you have a big break then, each time you go back, the likelihood is you’ve physically deteriorated – so the break just highlights the physical deterioration. It’s easier, psychologically, to experience gradual increasing difficulties over time, rather than perceive step changes in the decline of physical function. The problem then is, how do we do approach the ideal?

## Conclusions

Our study has shown that a full-scale RCT of AT, designed along frequentist lines, is not feasible in the UK alone. Any future studies should consider Bayesian approaches to achieve a statistically valid result with an achievable number of patients in a reasonable timeframe. Even then, the effect on motor function may not be very marked and a composite assessment also capturing individual patients well-being should be considered. We have demonstrated that AT has other benefits which are valued by people with DMD and physiotherapists. Two reviews and a conceptual framework [35, 36] offer guidance for selecting measures of participation which, we propose, should be considered as primary outcomes in future research.

Our study highlights, once again [37], the lack of basic information on the effectiveness, selection/prioritisation, dosage, and practicability of valid therapy protocols for stretches/exercises in people with DMD. Our treatment manuals offer a basis for a service, but more work is needed to understand how it can best be made personalised [38–41], developmentally appropriate [42] and coordinated. To ensure new models of provision meet the needs of stakeholders, further service development of the work should be co-produced [39, 43, 44], involving DMD family members, specialist and community physiotherapists, representatives of schools and/or teaching assistants, community AT pool providers and third sector organisations. Participation is currently inequitable, being determined by income and location [25, 45]. The UN Convention on the Rights of Persons with Disabilities (Articles 19, 25 and 30) [46] and, in the UK, The Children and Families Act 2014 (special educational and health provision reasonably required) [47, 48] provide an ethical and legal basis to guide co-production of new services. Where the capacity of parents and teaching assistants allows [49], physiotherapists can build capacity and share responsibility for the work of rehabilitation in the child, family and community. This could involve providing training and printed information on exercises that can be performed safely and effectively with their boys, as well as improved signposting to services [50–55].

Any future studies should consider alternative strategies such as Bayesian analytical approaches to deliver a statistically valid result with an achievable number of patients in a reasonable timeframe. To improve participation by NHS trusts in future trials, we recommend a review of excess treatment cost provision, early engagement of NHS England, consideration of refunding travel costs to study participants and randomisation by family and not individual.

## Additional files

Additional file 1: CONSORT. (DOCX 24 kb)

Additional file 2: Hydrotherapy manual. (PDF 136 kb)

Additional file 3: Land-based therapy manual. (PDG 256 kb)

## Abbreviations

6MWD: Six-Minute Walking Distance
ACTIVLIM: Activity limitations measure
CarerQoL: Carer quality of life
CHU9D: Child Health Utility 9D Index
CONSORT: Consolidated Standards of Reporting Trials
DMD: Duchenne muscular dystrophy
FVC: Forced vital capacity
IQR: Interquartile range
ITT: Intention to treat
LBT: Land-based therapy
MCID: Minimum clinically important difference
NSAA: North Star Ambulatory Assessment
SD: Standard deviation
TMG: Trial Management Group
TSC: Trial Steering Committee
