# Supplementary material for: Aquatic therapy for boys with Duchenne muscular dystrophy (DMD): an external pilot randomised controlled trial
Source: Pilot Feasibility Stud. 2017 Mar 27;3:16. doi: 10.1186/s40814-017-0132-0 (PMC5367005; doi:10.1186/s40814-017-0132-0)
Supplement: Supplementary file 2 — Hydrotherapy manual. (PDF 136 kb) [file 40814_2017_132_MOESM2_ESM.pdf]

Hydrotherapy

HydroDMD

Randomisation No. R _ _ / _ _ _

Stretches

Upper Limbs	Therapy prescription	Session Date __/__/__	Session Date __/__/__	Session Date __/__/__	Session Date __/__/__	Session Date __/__/__	Session Date __/__/__	Session Date __/__/__	Session Date __/__/__
**Elbow flexor stretch**<br>Subject position: Inclined standing or sitting with the shoulder abducted with a float in their hand.<br>Therapist position: Standing behind, to the side or in front of the subject stabilising the position of the upper arm.<br>*Action: Extend the elbow and then push the float down into the water. The position is held for the count of 5. The arm is then relaxed allowing it to move up in the water producing a stretch until limited by discomfort or anatomical restriction occurs.*<br><br>*Alternatives: Use a plinth, sit or lean against the wall without a float. Use turbulent drag as therapist moves subject. BC with float and momentum. Modified Bad Ragaz Patterns*<br><br>*Comment*	☐ Yes<br>☐ No								
**Wrist**<br>Subject position: Standing holding or hands flat on a small float<br>Therapist position: Stabilises the distal forearm if required<br>*Action: Flex the wrist with the forearm pronated and under the water.*<br><br>*Comment*	☐ Yes<br>☐ No								

Stretches

Upper Limbs	Therapy prescription	Session Date __/__/__	Session Date __/__/__	Session Date __/__/__	Session Date __/__/__	Session Date __/__/__	Session Date __/__/__	Session Date __/__/__	Session Date __/__/__
**Radio-ulnar Supination/Pronation**<br>Subject position: Arm in abduction, external rotation, supination, wrist extension and finger extension via positioning by therapist or against the poolside wall<br>Therapist position:<br>*Action: Move the trunk away from the arm (this also gives a pectoral stretch) to bat a ball etc. with opposite hand.*<br>*Comment*	☐ Yes<br>☐ No								
**Ai Chi uplifting**<br>In early stages supination and pronation can also be combined with BA shoulder medial rotation (arm up against back) and pushing floats under water. Also simple pronation/supination wearing a swim glove or using a paddle to reduce streamlining<br>*Comment*	☐ Yes<br>☐ No								
**Thumb and Finger stretches**<br>Need to combine with wrist and elbow as a long finger flexor stretch.<br>*Comment*	☐ Yes<br>☐ No								

Stretches

Lower Limbs	Therapy prescription	Session Date __/__/__	Session Date __/__/__	Session Date __/__/__	Session Date __/__/__	Session Date __/__/__	Session Date __/__/__	Session Date __/__/__	Session Date __/__/__
**Hip Flexor stretch**<br>Subject position: Standing facing the wall holding onto the rail they flex one knee against the wall to stabilise the pelvis. A float is placed on the other knee.<br>Therapist position: Stand behind the subject and stabilise the pelvis and trunk<br>*Action: Push the float down into the water. The position is held for the count of 5. The leg is then relaxed allowing it to move up in the water producing a stretch until limited by discomfort or anatomical restriction occurs.*<br><br>*Alternatives: Use a plinth or lean against the wall without a float. Use side lying with drag and modified Watsu® hold. Use modified Bad Ragaz patterns in supine. Use turbulent drag supported upright by the therapist and using a noodle or body band.*<br><br>*Comment*	☐ Yes<br>☐ No								
**ITB Hip abductors and external rotators**<br>Need to prevent too much knee flexion.<br>Adapted Watsu® in supported side lying.<br>Incorporate into half roll facilitating from legs, upper body and neck supported by floats.<br>Sitting to lying with drag and use of trunk side flexion and rotation, use of sandwich position to prevent overuse of hip flexors.<br>Rhythmic Stabilisations at trunk with rotations/side flexion – plank.<br><br>*Comment*	☐ Yes<br>☐ No								

Stretches

Lower Limbs	Therapy prescription	Session Date __/__/__	Session Date __/__/__	Session Date __/__/__	Session Date __/__/__	Session Date __/__/__	Session Date __/__/__	Session Date __/__/__	Session Date __/__/__
Knee Flexor stretch (1)<br>Subject position: Sitting on a step, plinth or submerged stool with the knee extended and stabilising their thigh with their hand. A float is placed on the ankle.<br>Therapist position: The therapist may need to stabilise the thigh.<br>*Action: Push the float down into the water. The position is held for the count of 5. The leg is then relaxed allowing extensions to occur until limited by discomfort or anatomical restriction occurs.*<br>*Comment*	☐ Yes<br>☐ No								
Knee Flexor stretch (2)<br>Subject position: Squatting with their back against the wall and one leg extended out in front of them. A float on the knee.<br>Therapist position:<br>*Action: Raise the leg as far as possible without flexing the knee. The positon is held for a count of 5 and the subject then attempts to raise the leg further until limited by discomfort or anatomical restriction occurs.*<br><br>*Alternatives: Use a plinth, sit on step or sunken stool/seat or therapist. without a float. Use side lying with drag and modified Watsu® hold. Use modified Bad Ragaz patterns in supine and sitting. Use turbulent drag supported upright by the therapist and using a noodle or body band. Use buoyancy by supported sitting and lowering body down into water with leg outstretched.*<br>*Push offs from the wall*<br>*Comment*	☐ Yes<br>☐ No								

Stretches

Lower Limbs	Therapy prescription	Session Date __/__/__	Session Date __/__/__	Session Date __/__/__	Session Date __/__/__	Session Date __/__/__	Session Date __/__/__	Session Date __/__/__	Session Date __/__/__
Ankle and Foot stretches<br>Subject position: Sitting on a plinth, step or submersed stool with the knee slightly flexed.<br>Therapist position: Facing the subject. Hand under the heel and forearm along the plantar aspect of the foot.<br>*Action: Stretches against the wall. Incorporate into modified Bad Ragaz patterns supine and sitting. Sandwich support to maintain hip extension whilst rotating and/or side flexing the trunk. Stand on a noodle and allow foot to come up.*<br>*Comment*	☐ Yes<br>☐ No								

Stretches

Trunk and Neck	Therapy prescription	Session Date __/__/__	Session Date __/__/__	Session Date __/__/__	Session Date __/__/__	Session Date __/__/__	Session Date __/__/__	Session Date __/__/__	Session Date __/__/__
Neck stretches<br>Subject position: In supine float<br>Therapist position: Supporting the head with their hands.<br>*Action: The therapist flexes the neck by moving the head and elongating the neck.*<br><br>*Comment*	☐ Yes<br>☐ No								
Trunk Stretches Rotation<br>Subject position: Seated and immersed<br>Therapist position: May need to stabilise the pelvis.<br>*Action: The subject reaches with both hands on a float or supported with an inflatable ring. The therapist assists them to rotate the trunk. The position is held for the count of 5. The legs are then relaxed allowing them to move up in the water producing a stretch until limited by discomfort or anatomical restriction occurs.*<br><br>*Comment*	☐ Yes<br>☐ No								

Stretches

Trunk and Neck	Therapy prescription	Session Date __/__/__	Session Date __/__/__	Session Date __/__/__	Session Date __/__/__	Session Date __/__/__	Session Date __/__/__	Session Date __/__/__	Session Date __/__/__
Trunk Stretches Side Flexion<br>Subject position: Facing the wall holding the rail. The knees are then either flexed or extended depending upon the depth of the pool, the hips must remain extended.<br>Therapist position: Stabilising the upper trunk.<br>*Action: The subject allows the legs to move up in the water. They then push them down into the water. The positon is held for the count of 5. The legs are then relaxed allowing them to move up in the water producing a stretch until limited by discomfort or anatomical restriction occurs.*<br><br>*Seaweeding and trunk elongation in supine float and an upright position, include the shoulder, BC with drag for both side flexion and rotation, include pectoral stretch.*<br><br>*Comment*	☐ Yes<br>☐ No								

Muscle Training: Stage One (early)

Upper Limbs	Therapy prescription	Session Date __/__/__	Session Date __/__/__	Session Date __/__/__	Session Date __/__/__	Session Date __/__/__	Session Date __/__/__	Session Date __/__/__	Session Date __/__/__
Shoulder Abduction<br>Use buoyancy counterbalanced position or standing and increase speed, then add a bat.<br>Penguin arms with speed whilst standing or sitting on a woggle or kickboard.<br>*Comment*	☐ Yes<br>☐ No								
Shoulder Flexion and Extension<br>(fingers open or closed to affect drag and streamlining)<br>Use standing position and increase speed, then add a bat with forward and backward walking<br>*Comment*	☐ Yes<br>☐ No								
Elbow Flexion and Extension<br>Use standing position and increase speed, then add a bat or swim glove, pushing float down.<br>*Comment*	☐ Yes<br>☐ No								

Muscle Training: Stage One (early)

Upper Limbs	Therapy prescription	Session Date __/__/__	Session Date __/__/__	Session Date __/__/__	Session Date __/__/__	Session Date __/__/__	Session Date __/__/__	Session Date __/__/__	Session Date __/__/__
Wrist and fingers All movements<br>In standing or sitting on a plinth, step or submersed stool. Keep the arm by the side with the elbow flexed to about 90 degrees. Increase speed of movements or use a bat.<br><br>*Comment*	☐ Yes<br>☐ No								

Randomisation No. R _ _ / _ _ _

Muscle Training: Stage One (early)

Lower Limbs	Therapy prescription	Session Date __/__/__	Session Date __/__/__	Session Date __/__/__	Session Date __/__/__	Session Date __/__/__	Session Date __/__/__	Session Date __/__/__	Session Date __/__/__
Hip Extension<br>In supine float (preventing the trunk from extending). Standing increase speed.<br><br>*Comment*	☐ Yes<br>☐ No								
Hip Adduction<br>Supine float or standing, increase speed and cross leg side step.<br><br>*Comment*	☐ Yes<br>☐ No								
Knee Extension<br>Incorporate into hip extension exercise in standing. Walking backwards.<br><br>*Comment*	☐ Yes<br>☐ No								
Ankle and Foot All movements<br>Incorporate into the hip extension exercise in standing. Increase speed and use a shorty flipper, metacentric and standing work, push offs from the wall and stepping onto and over a submerged step.<br><br>*Comment*	☐ Yes<br>☐ No								

Muscle Training: Stage One (early)

Trunk	Therapy prescription	Session Date __/__/__	Session Date __/__/__	Session Date __/__/__	Session Date __/__/__	Session Date __/__/__	Session Date __/__/__	Session Date __/__/__	Session Date __/__/__
Trunk Rotation<br>In supine float with the knees flexed to approximately 90°. Increase the speed of movement/deflate the float around the ankle. Resisted rolling by therapist movement away from the direction of roll, creating therapist drag as transitions between lying and sitting using truncal rotation and side flexion with hips in extension, using metacentric principles.<br><br>*Comment*	☐ Yes<br>☐ No								
Trunk Side Flexion<br>Alternatively in supine float holding onto the rail with a float around the ankle increase the speed of movement/deflate the float around the ankle. Tick tock standing with floats in each hand.<br><br>*Comment*	☐ Yes<br>☐ No								

Muscle Training: Stage Two

Upper Limbs	Therapy prescription	Session Date __/__/__	Session Date __/__/__	Session Date __/__/__	Session Date __/__/__	Session Date __/__/__	Session Date __/__/__	Session Date __/__/__	Session Date __/__/__
Shoulder<br>In supine float with the body supported with the relevant flotation (with a float above the wrist), supine float or lying supine on a plinth.<br><br>*Comment*	☐ Yes<br>☐ No								
Shoulder Rotation<br>In standing with their arm by their side and elbow flexed to 90°.<br><br>*Comment*	☐ Yes<br>☐ No								
Elbow Flexion and Extension<br>In supine float with the body supported with the relevant flotation (with a float above the wrist) supine float or lying supine on a plinth or sitting with speed.<br><br>*Comment*	☐ Yes<br>☐ No								
Wrist and Fingers All movements<br>In standing or sitting on a plinth, step or submersed stool, use glove initially for resistance as appropriate, play piano, floating xylophone, floating drums, watering cans, sieves.<br><br>*Comment*	☐ Yes<br>☐ No								

Muscle Training: Stage Two

Lower Limbs	Therapy prescription	Session Date __/__/__	Session Date __/__/__	Session Date __/__/__	Session Date __/__/__	Session Date __/__/__	Session Date __/__/__	Session Date __/__/__	Session Date __/__/__
Hip Flexion and Extension<br>Floating on their side with support from the physiotherapist. Standing with speed. Walking forwards and backwards. Ai Chi gathering and freeing. Free floating with a noodle or body band.<br>*Comment*	☐ Yes<br>☐ No								
Hip Adduction<br>In supine float holding onto the rail with the body supported with the relevant flotation with a float around the ankle with speed. Cross leg stepping up and down incline in pool (if there is one). Standing with speed. Diving stick races (kick water just in front to make them move).<br>*Comment*	☐ Yes<br>☐ No								
Knee Flexion and Extension<br>Floating on their side holding onto the rail or with support from therapist or on plinth with a float around the ankle. The physiotherapist stabilises the hip. Sitting with a body/leg band around legs to keep in neutral position (out of abduction). Cycle legs, motor boats and speed boats, quarter turn prone.<br>*Comment*	☐ Yes<br>☐ No								
Ankle and Foot All movements<br>Incorporated into the knee movements.<br>*Comment*	☐ Yes<br>☐ No								

Muscle Training: Stage Two

Trunk	Therapy prescription	Session Date __/__/__	Session Date __/__/__	Session Date __/__/__	Session Date __/__/__	Session Date __/__/__	Session Date __/__/__	Session Date __/__/__	Session Date __/__/__
Metacentric Effects<br>Stand, sitting or supine float – change shape or immersed surface on one side – splash, push ball, lifts one hand out of water, different games that achieve the above in different starting positions.<br>*Comment*	☐ Yes<br>☐ No								
Trunk Rotation<br>Sit or lying to sitting, the therapist steps forward or back to facilitate or resist the movement as they reach to the opposite shoulder. The subject faces the wall holding the rail. The knees are then flexed to approximately 90° and the trunk rotated to left and right.<br>*Comment*	☐ Yes<br>☐ No								
Trunk Side Flexion<br>In supine float holding onto the rail. The physiotherapist stabilises the upper body if necessary. Drag effect through upper limb activity, BC at pelvis, keep hips extended and use snaking, one side only and block pelvis if scoliosis/pelvic obliquity present.<br>*Comment*	☐ Yes<br>☐ No								
