# Supplementary material for: Aquatic therapy for boys with Duchenne muscular dystrophy (DMD): an external pilot randomised controlled trial
Source: Pilot Feasibility Stud. 2017 Mar 27;3:16. doi: 10.1186/s40814-017-0132-0 (PMC5367005; doi:10.1186/s40814-017-0132-0)
Supplement: Supplementary file 3 — Land-based therapy manual. (PDG 256 kb) [file 40814_2017_132_MOESM3_ESM.pdf]

HydroDMD

Land Based Stretches and Exercises

FOR OFFICE USE ONLY

Randomisation No. R | | | / | | |

Week 1 start date

i.e. the date you first started doing the stretches and exercises recorded in this booklet.

d	d	m	m	y	y	y	y

Stretches

Upper Limbs	Therapy prescription	Week 1: How many days?	Week 2: How many days?	Week 3: How many days?	Week 4: How many days?
Elbow Extension combined with supination<br>Subject position: supine or sitting with back and feet supported. Arm to be stretched in full possible extension with hand supinated. Therapist position: standing/sitting topside of arm to be stretched. One hand cupping posterior aspect of elbow joint, other hand on anterior surface of wrist.<br>*Action: holding upper arm still with hand at elbow joint, put arm on stretch into maximum comfortable extension. Ensure elbow does not go into hyper extension. Hold for a count of 5, repeat 5 times each arm.*<br>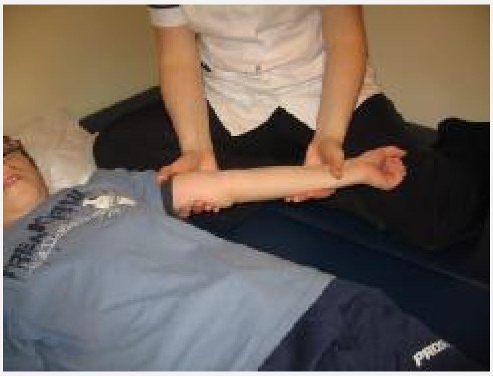<br>Research physiotherapist comment:<br>Changed by community physiotherapist?: ☐ Yes ☐ No<br>Community physiotherapist comment:<br>*Comment*	☐ Yes<br>☐ No				

For information about this trial please contact the Study Manager, Chin Maguire:
Tel. 0114 222 0717 or email: c.maguire@sheffield.ac.uk.
Or see the website http://www.sheffield.ac.uk/scharr/sections/dts/ctru/hydrodmd

Stretches

Upper Limbs	Therapy prescription	Week 1: How many days?	Week 2: How many days?	Week 3: How many days?	Week 4: How many days?
**Wrist, thumb and finger stretches**<br>Subject position: supine or sitting with back and feet supported. Elbow flexed approximately 15°, forearm supinated.<br>Therapist position: sitting or standing beside child on side to be stretched. One hand supporting forearm, other hand holding child's hand in the palm.<br>*Action: with hand that's in palm, hold wrist in maximum extension, and extend fingers and thumb. Hold for a count of 5, repeat 5 times each hand. If therapist cannot hold fingers and thumb with one hand, ensure child's elbow is supported and use 2nd hand to extend thumb.*<br>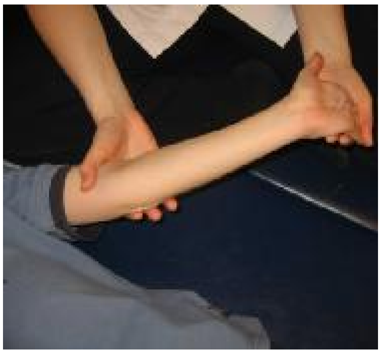<br>Research physiotherapist comment:<br>Changed by community physiotherapist?: ☐ Yes ☐ No<br>Community physiotherapist comment:<br>*Comment*	☐ Yes<br>☐ No				

Stretches

Lower Limbs	Therapy prescription	Week 1: How many days?	Week 2: How many days?	Week 3: How many days?	Week 4: How many days?
Hip Extension<br>1. Prone:<br>Subject position: child lying face down.<br>Therapist position: standing or kneeling beside child.<br>*Action: hold pelvis with one hand to stop it rotating. Extend leg with knee held straight. Hold for count of 5, repeat 5 times each leg.*<br>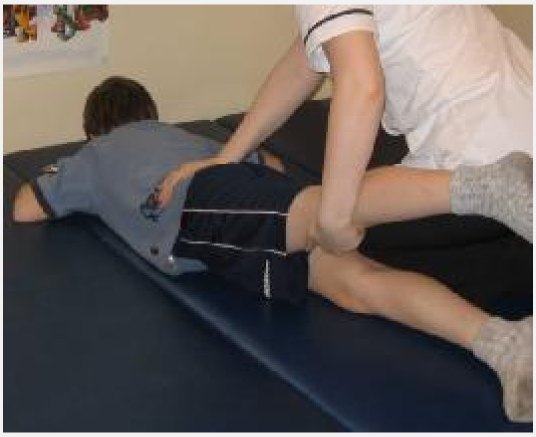<br>Research physiotherapist comment:<br>Changed by community physiotherapist?: ☐ Yes ☐ No<br>Community physiotherapist comment:<br>*Comment*	☐ Yes<br>☐ No				

Stretches

Lower Limbs	Therapy prescription	Week 1: How many days?	Week 2: How many days?	Week 3: How many days?	Week 4: How many days?
Hip Extension<br>2. Supine<br>Subject position: with child lying supine on a plinth with legs over the edge.<br>Therapist position: standing beside the child.<br>*Action: flex one knee up towards the chest, hold the other leg downwards towards the floor. Hold for a count of 5, repeat 5 times each leg.*<br>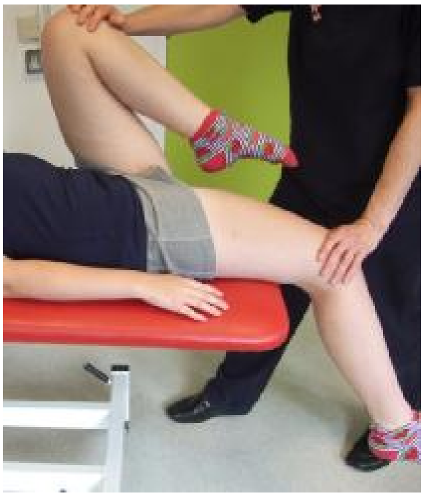<br>Research physiotherapist comment:<br><br>Changed by community physiotherapist?: ☐ Yes ☐ No<br>Community physiotherapist comment:<br><br>*Comment*	☐ Yes<br>☐ No				

HydroDMD

Land Based Stretches and Exercises

FOR OFFICE USE ONLY

Randomisation No. R | | / | |

Stretches

Lower Limbs	Therapy prescription	Week 1: How many days?	Week 2: How many days?	Week 3: How many days?	Week 4: How many days?
ITB stretch<br>1. Prone<br>Subject position: child lying face down.<br>Therapist position: standing or kneeling beside child at the opposite side to the ITB to be stretched.<br>*Action: hold pelvis with one hand to stop it rotating. Extend leg with knee held straight, adduct across the other leg, keeping the knee straight. . Hold for count of 5, repeat 5 times each leg.*<br>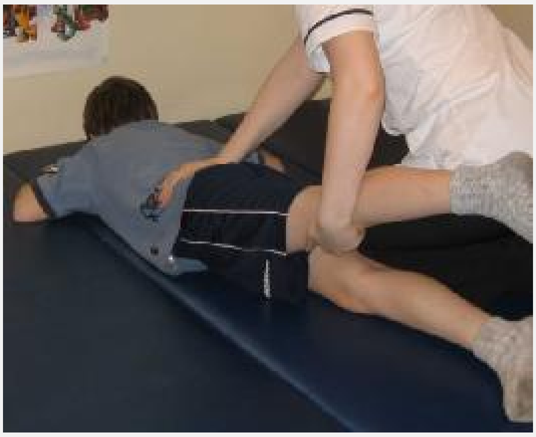<br>Research physiotherapist comment:<br>Changed by community physiotherapist?: ☐ Yes ☐ No<br>Community physiotherapist comment:<br>*Comment*	☐ Yes<br>☐ No				

Stretches

Lower Limbs	Therapy prescription	Week 1: How many days?	Week 2: How many days?	Week 3: How many days?	Week 4: How many days?
ITB stretch<br>2. Lateral<br>Child position: side lying on plinth towards the edge.<br>Therapist position: standing behind the child, holding the pelvis with one hand and the upper leg with the other.<br>*Action: extend, externally rotate and adduct the leg behind the other, off the plinth, keeping the knee straight. Hold for a count of 5, repeat 5 times each leg.*<br>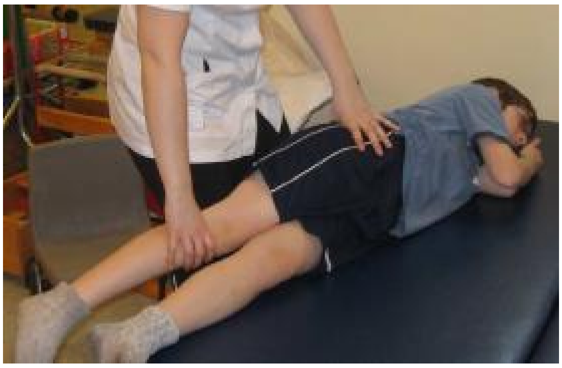<br>Research physiotherapist comment:<br>Changed by community physiotherapist?: ☐ Yes ☐ No<br>Community physiotherapist comment:<br>*Comment*	☐ Yes<br>☐ No				

Stretches

Lower Limbs	Therapy prescription	Week 1: How many days?	Week 2: How many days?	Week 3: How many days?	Week 4: How many days?
Knee extension<br>Subject position: lying supine.<br>Therapist position: standing or kneeling beside the child on the same side as the knee to be stretched. One hand just above the knee, second hand holding the foot.<br>*Action: lift lower leg to extend the knee as much as possible keeping the foot dorsiflexed to ensure a good stretch. Hold for a count of 5, repeat 5 times each leg.*<br>*Ensure the knee does not hyperextend.*<br>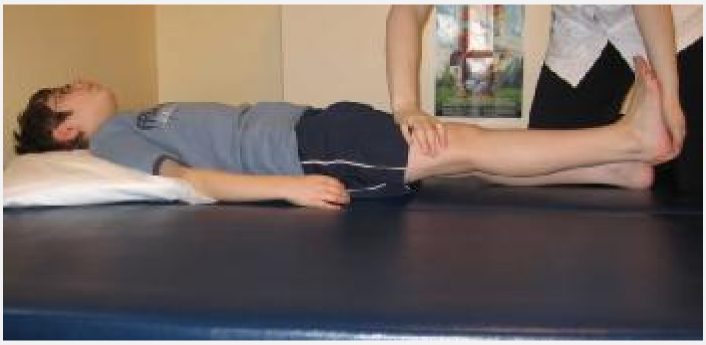<br>Research physiotherapist comment:<br><br>Changed by community physiotherapist?: ☐ Yes ☐ No<br>Community physiotherapist comment:<br><br>*Comment*	☐ Yes<br>☐ No				

Stretches

Lower Limbs	Therapy prescription	Week 1: How many days?	Week 2: How many days?	Week 3: How many days?	Week 4: How many days?
Ankle and Foot Stretches<br>Dorsiflexion<br>Subject position: lying supine.<br>Therapist position: standing or kneeling beside the child on the same side as the ankle to be stretched. Hold the leg just above the knee and the other hand cupping the heel, and up the foot<br>*Action: stretch the Achilles tendon, bringing the toes up towards the body. Hold for a count of 5, repeat 5 times each leg.*<br>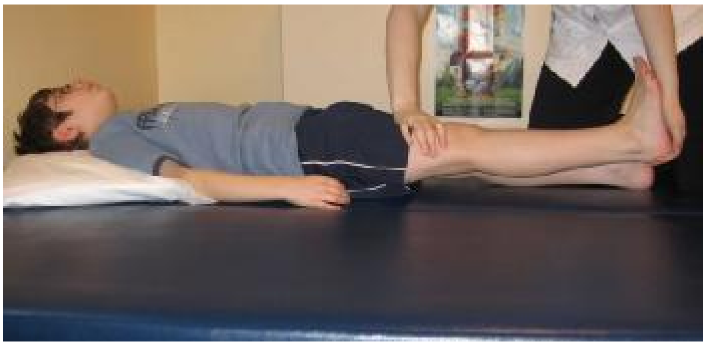<br>Research physiotherapist comment:<br>Changed by community physiotherapist?: ☐ Yes ☐ No<br>Community physiotherapist comment:<br>*Comment*	☐ Yes<br>☐ No				

Stretches

Trunk and Neck	Therapy prescription	Week 1: How many days?	Week 2: How many days?	Week 3: How many days?	Week 4: How many days?
Neck stretches<br>1. Side flexion<br>Subject position: supine lying.<br>Therapist position: to the side of the child, holding the child's head.<br>*Action: move the ear towards the shoulder on the same side. Hold for a count of 5, repeat 5 times to each side.*<br>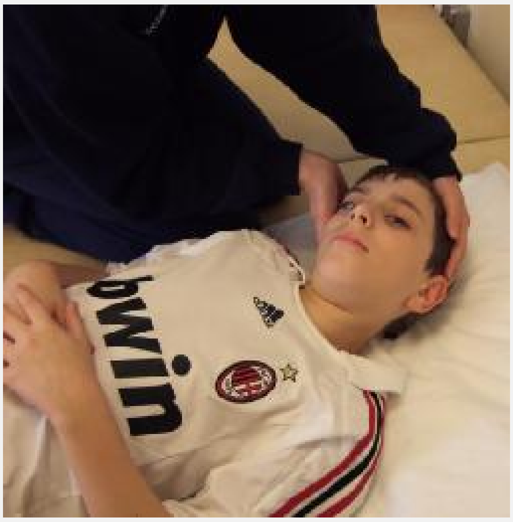<br>Research physiotherapist comment:<br>Changed by community physiotherapist?: ☐ Yes ☐ No<br>Community physiotherapist comment:<br>*Comment*	☐ Yes<br>☐ No				

Stretches

Trunk and Neck	Therapy prescription	Week 1: How many days?	Week 2: How many days?	Week 3: How many days?	Week 4: How many days?
Neck stretches<br>2. Rotation<br>Subject position: supine lying.<br>Therapist position: to the side of the child, holding the child's head.<br>*Action: turn the head towards the shoulder on the same side. Hold for a count of 5, repeat 5 times to each side.*<br>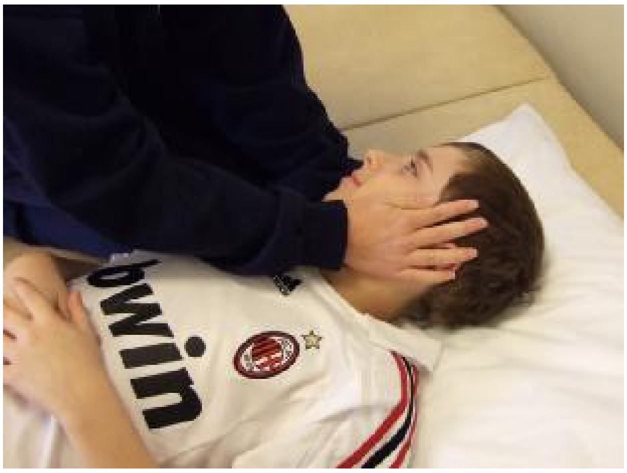<br>Research physiotherapist comment:<br>Changed by community physiotherapist?: ☐ Yes ☐ No<br>Community physiotherapist comment:<br>*Comment*<br>Not included – sub-talar joint, trunk stretches	☐ Yes<br>☐ No				

Strengthening: using gravity as the resistance

Upper Limbs	Therapy prescription	Week 1: How many days?	Week 2: How many days?	Week 3: How many days?	Week 4: How many days?
Shoulder Abduction<br><br>Sitting in chair without arms or standing: lift arms slowly out to sides until they reach 90. Hold for a count of 3. Relax them down. Repeat 5 times.<br><br>Research physiotherapist comment:<br><br>Changed by community physiotherapist?: ☐ Yes ☐ No<br>Community physiotherapist comment:<br><br>*Comment*	☐ Yes<br>☐ No				
Shoulder Flexion<br><br>Sitting in a chair or standing: lift arms slowly forwards to 180. Hold for a count of 3. Relax them down. Repeat 5 times.<br><br>Research physiotherapist comment:<br><br>Changed by community physiotherapist?: ☐ Yes ☐ No<br>Community physiotherapist comment:<br><br>*Comment*	☐ Yes<br>☐ No				
Shoulder Extension<br><br>Lying prone, with arms by sides, and keeping them into sides, lift arms slowly as far possible backwards into extension, hold for a count of 3. Relax them down. Repeat 5 times.<br><br>Research physiotherapist comment:<br><br>Changed by community physiotherapist?: ☐ Yes ☐ No<br>Community physiotherapist comment:<br><br>*Comment*	☐ Yes<br>☐ No				

Strengthening: using gravity as the resistance

Upper Limbs	Therapy prescription	Week 1: How many days?	Week 2: How many days?	Week 3: How many days?	Week 4: How many days?
Elbow Extension<br><br>Lying supine: lift arm to 90 degrees of flexion, and adducted into body. Flex elbow so hand is touching opposite shoulder. Lift hand slowly up until elbow is fully extended. Hold for count of 3. Relax hand back down to shoulder. Repeat 5 times.<br><br>Research physiotherapist comment:<br><br>Changed by community physiotherapist?: ☐ Yes ☐ No<br>Community physiotherapist comment:<br><br>*Comment*	☐ Yes<br>☐ No				
Wrist and finger Extension<br><br>Sitting with arm supported on the arm of a chair or with forearm on table, so that the hands are unsupported and wrist fully flexed. Pull the wrist and fingers back slowly into maximum possible extension. Hold for a count of 3. Repeat 5 times.<br><br>Research physiotherapist comment:<br><br>Changed by community physiotherapist?: ☐ Yes ☐ No<br>Community physiotherapist comment:<br><br>*Comment*	☐ Yes<br>☐ No				

Strengthening: using gravity as the resistance

Lower Limbs and Trunk	Therapy prescription	Week 1: How many days?	Week 2: How many days?	Week 3: How many days?	Week 4: How many days?
Hip Extension<br><br>Standing with trunk flexed over a plinth, height adjusted to waist level. Lift one leg slowly backwards as high as possible, keeping the knee straight. Hold for a count of 3. Relax down. Repeat 5 times.<br><br>Alternative position: standing holding on to the back of a chair. Keep the trunk upright and lift one leg slowly backwards, keeping the trunk upright and knee straight throughout the movement.<br><br>Research physiotherapist comment:<br><br>Changed by community physiotherapist?: ☐ Yes ☐ No<br>Community physiotherapist comment:<br><br>*Comment*	☐ Yes<br>☐ No				
Hip Abduction<br><br>Side lying: lift the leg slowly, with the knee extended, to at least 30° keeping the leg in line with the body. Relax down. Hold for a count of 5, repeat 5 times each leg.<br><br>Research physiotherapist comment:<br><br>Changed by community physiotherapist?: ☐ Yes ☐ No<br>Community physiotherapist comment:<br><br>*Comment*	☐ Yes<br>☐ No				

Strengthening: using gravity as the resistance

Lower Limbs and Trunk	Therapy prescription	Week 1: How many days?	Week 2: How many days?	Week 3: How many days?	Week 4: How many days?
Knee Extension (1)<br><br>Sitting with the foot not in contact with the floor. Raise the leg slowly until the knee is straight. Hold for a count of 5. Relax down. Repeat 5 times each leg.<br><br>Research physiotherapist comment:<br><br>Changed by community physiotherapist?: ☐ Yes ☐ No<br>Community physiotherapist comment:<br><br>*Comment*	☐ Yes<br>☐ No				
Knee Extension (2)<br><br>Lying supine with the knee flexed up to 45°, foot in contact with the surface. Extend the knee until the leg is straight. Hold for a count of 5. Relax down. Repeat 5 times each leg.<br><br>Research physiotherapist comment:<br><br>Changed by community physiotherapist?: ☐ Yes ☐ No<br>Community physiotherapist comment:<br><br>*Comment*	☐ Yes<br>☐ No				
Ankle and foot<br><br>Sitting: with the foot off the floor to allow full plantar flexion with the toes not in contact with the floor. Pull foot up as far as possible. Hold for a count of 5. Relax down. Repeat 5 times each foot.<br><br>Research physiotherapist comment:<br><br>Changed by community physiotherapist?: ☐ Yes ☐ No<br>Community physiotherapist comment:<br><br>*Comment*	☐ Yes<br>☐ No				

Strengthening: using gravity as the resistance

Lower Limbs and Trunk	Therapy prescription	Week 1: How many days?	Week 2: How many days?	Week 3: How many days?	Week 4: How many days?
Trunk Extension<br><br>Child lying prone, hands on buttocks, extend head, shoulders and trunk as high as possible. Hold for a count of 5. Relax down, repeat 5 times.<br><br>Research physiotherapist comment:<br><br>Changed by community physiotherapist?: ☐ Yes ☐ No<br>Community physiotherapist comment:<br><br>*Comment*	☐ Yes<br>☐ No				
Trunk Side Flexion<br><br>Child side lying with arm on trunk. Reach arm down leg lifting trunk sideways off plinth. Hold for a count of 5, relax down. Repeat 5 times each side.<br><br>Research physiotherapist comment:<br><br>Changed by community physiotherapist?: ☐ Yes ☐ No<br>Community physiotherapist comment:<br><br>*Comment*	☐ Yes<br>☐ No				
